# Breast cancer quality of care in Syria: screening, diagnosis, and staging

**DOI:** 10.1186/s12885-023-11740-2

**Published:** 2023-12-14

**Authors:** Fouad Nahhat, Modar Doyya, Kareem Zabad, Tarek Abo Laban, Hasan Najjar, Maher Saifo, Firas Badin

**Affiliations:** 1 Faculty of Medicine, Damascus University, Damascus, Syrian Arab Republic; 2 Medical Director for Oncology Research, Baptist Health Lexington, Lexington, KY USA

**Keywords:** Breast cancer, Quality of care, Syria, Syrian war, Cancer screening, Early detection, Stage at diagnosis, Method of diagnosis

## Abstract

**Background:**

The Syrian decade-long war has severely affected the healthcare system, including almost vanishing cancer screening practices, war-destroyed medical facilities, and lack of continuous medical education. This study aims to present data on the affected breast cancer screening practices, methods of diagnosis, and stages distribution in Syria.

**Methods:**

Medical charts of breast cancer patients treated at Albairouni University Hospital between January 2019 and May 2022 were retrospectively reviewed. Eligible patients were women diagnosed with primary breast cancer. Exclusion criteria included females receiving neoadjuvant chemotherapy and incomplete charts. Data regarding the patient’s age, city of residence, marital status, number of children, smoking habits, method of diagnosis, tumor size (T), lymph nodes (N), and distal metastasis (M) were collected. We used Microsoft Excel and Statistical Package for the Social Sciences (SPSS) to analyze data. Descriptive methodology (frequency [n], percentage) was used.

**Results:**

The number of charts reviewed was 4,500. The number of remaining charts after applying the exclusion criteria was 2,367. The mean age was 51.8 (SD = 11.3). More than half of the patients (58.3%) came from outside Damascus -where the hospital is located- and its suburbs. Less than 5% of the population detected cancer by screening mammography. Only 32.4% of patients were diagnosed by a biopsy, while surgical procedures (lumpectomy and mastectomy) were used to diagnose 64.8% of the population. At the time of diagnosis, only 8% of patients presented with local-stage disease (stages 0 & I), 73% had a regional disease (stages II & III), and 19% had metastatic breast cancer (stage IV).

**Conclusion:**

Our retrospective chart review analysis is the first comprehensive review in Syria for female breast cancer patients. We found a significant low percentage of patients diagnosed based on a screening mammogram, much higher surgical biopsies rather than a simple imaging-guided biopsy, and much lower than the national average of early-stage disease. Our alarming findings can serve as the base for future strategies to raise the population’s health awareness, create more serious national screening campaigns, and adopt a multidisciplinary approach to the disease in Syria.

## Background

Female breast cancer exceeded lung cancer as the most commonly diagnosed cancer worldwide in 2020, with an estimated incidence of 2.3 million new cases, making up 11.7% of all cancer cases. It is the fifth leading cause of cancer death worldwide, with 685,000 deaths in 2020. On the other hand, it is ranked first among fatal cancers in women [1].

Breast cancer has a relatively lower incidence but higher mortality rates in Western Asian countries compared with other regions such as Australia, Europe, and Northern America [1]. The higher mortality rates have been linked to various potential reasons, such as the lack of screening programs, substandard diagnosis and treatment, low socioeconomic level, and poor health awareness, including low awareness of breast cancer symptoms that, in turn, leads to delays in seeking medical care [2–5]. Other factors contributing to the hesitancy in seeking early medical help in Western Asian Countries include conflict-related inaccessibility to medical facilities and personal psychosocial factors such as shyness [5].

The disease stage at diagnosis is one of the most important prognostic factors in breast cancer [6]. The advanced stage at diagnosis is associated with poor prognosis and a lower survival rate [7].

Community-wide breast cancer screening programs help in the early detection of the disease, which in turn lowers mortality rates through effective treatment. Even though screening programs can result in some overdiagnosis [8], women who participated in these programs have a 60% and 47% lower risk of dying from the disease within ten years and 20 years after diagnosis, respectively, in comparison with those who did not enroll in screening programs [9].

The American Cancer Society recommends annual screening for women aged 45 to 54 and biennial screening for those aged ≥ 55 years. Women aged 40 to 44 years have the opportunity to screen themselves annually, and women should continue screening as long as they are in a good healthy state and have ≥ 10 years of life expectancy [10].

The Syrian decade-long war and its consequences had a negative impact on the Syrian healthcare system, including screening practices. With a lack of access to screening programs, oncology patients are presenting with advanced-stage malignancies, including breast cancer patients [11]. Moreover, the lack of studies on the disease quality of care and statistics in Syria necessitated our research paper, as it is the first retrospective analysis to go through these topics.

This study aims to present data on the current landscape of breast cancer care in Syria, in particular, the current screening practices among patients, methods of diagnosis, and current stages distribution. The ultimate goal is to provide a base for future strategies for improving breast cancer care in Syria.

## Methods

An ethical approval from The Scientific Research Ethics Committee of Damascus University was obtained (approval number: 3567). Informed consent was obtained from patients on the administration to the hospital.

Patients eligible for the study were women of all ages who were diagnosed with primary breast cancer. Exclusion criteria included females receiving neoadjuvant chemotherapy and charts with insufficient information for determining the stage.

Paper medical charts for patients treated at Al-Bairouni University Hospital in Harasta from January 2019 to May 2022, a total of 4,500 charts, were retrospectively reviewed. The number of remaining charts after applying the exclusion criteria was 2,367.

The patient’s gender, age at diagnosis, city of residency, marital status, number of children, smoking habits, method of diagnosis (biopsy/surgical procedure), primary tumor size (T), pathologic lymph nodes (N)- from the surgical or pathological report- and distal metastasis (M) -confirmed by radiological reports- were collected. The tumor stage was determined depending on The American Joint Committee on Cancer (AJCC) TNM system (8th edition) [12].

Study data were analyzed with Microsoft Office Excel 2019 and IBM Statistical Package for the Social Sciences (SPSS) v26. Descriptive methodology (frequency [n], percentage) was used. The chi-square test was used to determine statistical significance.

## Results

**Fig. 1:**
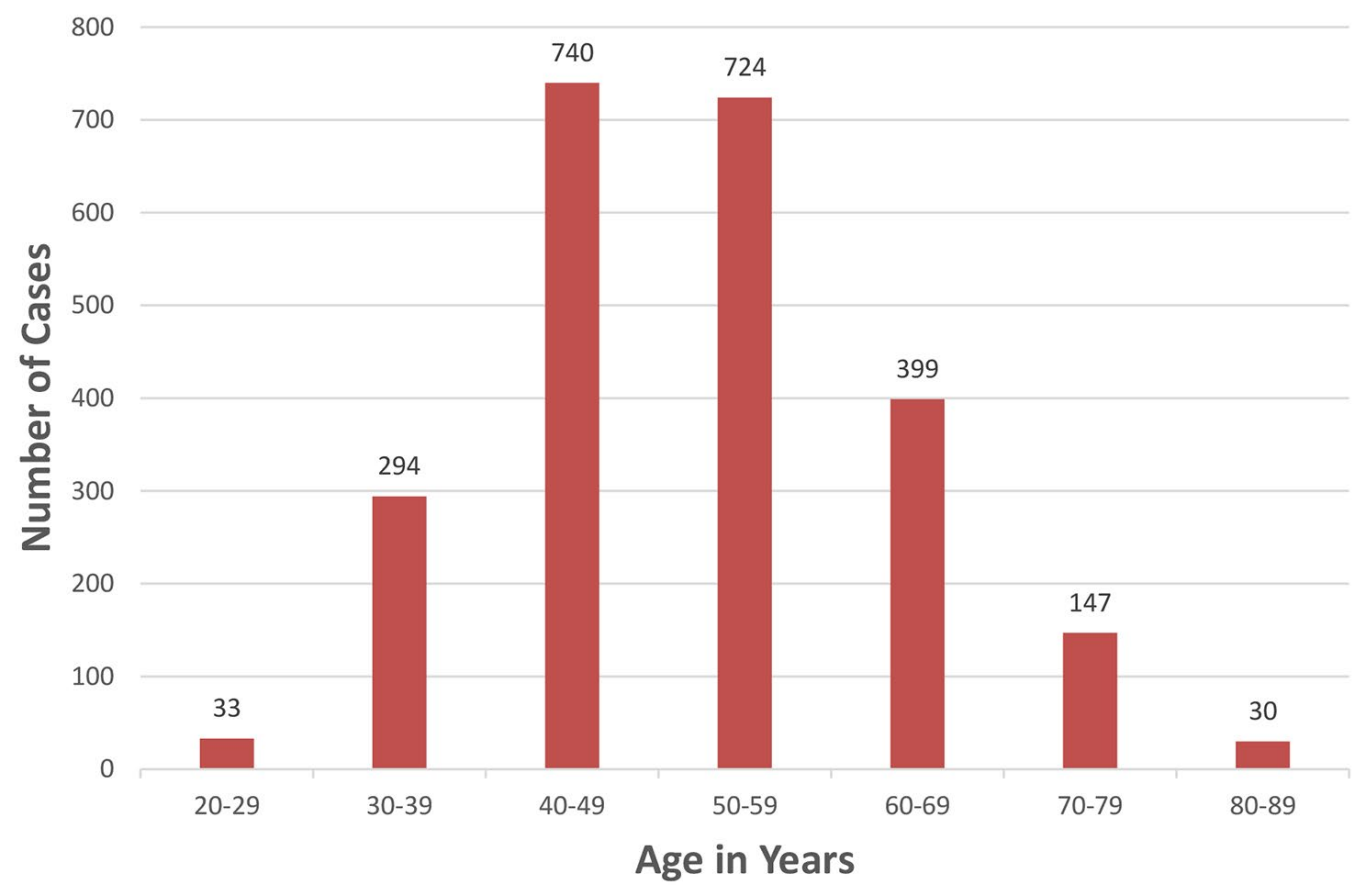
Age distribution of breast cancer patients in the sample

**Fig. 2:**
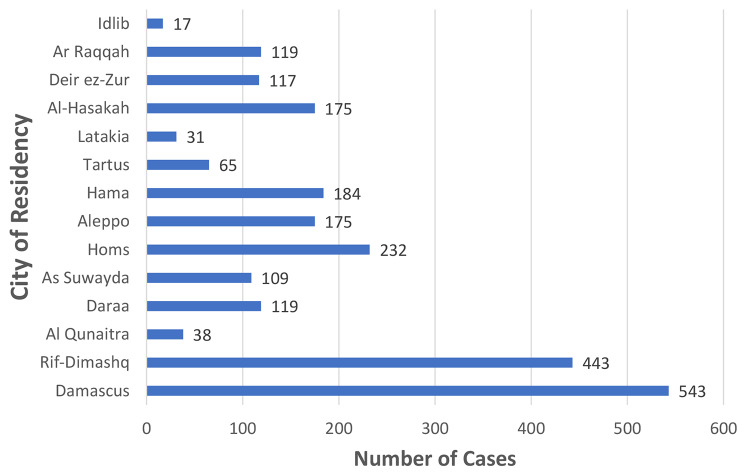
Geographical distribution of breast cancer patients treated at Albairouni University Hospital in the period between 2019 & 2022

A total of 2,367 patients were included in our study. The majority of them were non-smoker married housewives (Table 1). Patients’ ages ranged from 22 to 87 years. The median age was 51 years (Fig. 1). Noteworthily, more than half the patients (1,381) came from outside Damascus –where the hospital is- and its suburbs (Fig. 2).

**Table 1:** General characteristics of the sample

Characteristics	Count (n) [N = 2367]	Percentage (%)
**Age (years)**		
Median	51.448	-
Range	22–87	-
**Occupation**		
Had an Occupation	422	17.8
Housewife	1923	81.2
Missing	22	0.9
**Marital Status**		
Married	2132	90.1
Single	207	8.7
Missing	28	1.2
**Residency**		
Damascus & Rif-Dimashq	986	41.7
Other governorate	1381	58.3
**Habits**		
Smoker	431	18.2
Non smoker	1805	76.3
**Number of Children**		
0	182	7.7
1–3	827	34.9
4–6	791	33.4
7–9	256	10.8
≥10	76	3.2
Missing	235	9.9

Only 5% of the population detected cancer by screening mammography. In contrast, 95% had not enrolled in screening programs prior to diagnosis. Moreover, disease diagnosis in 1,533 patients (64.8%) was made by surgical procedures (lumpectomy and mastectomy) rather than a simple imaging-guided biopsy (Table 2).

**Table 2:** Method of diagnosis & disease detection

	Count (n) [N = 2367]	Percentage (%)
**Method of Diagnosis**		
Surgical Procedure	1533	64.8
Biopsy	766	32.4
Missing	68	2.8
**Disease Detection**		
After Feeling Symptoms	2248	95
By Screening Methods	119	5

**Fig. 3:**
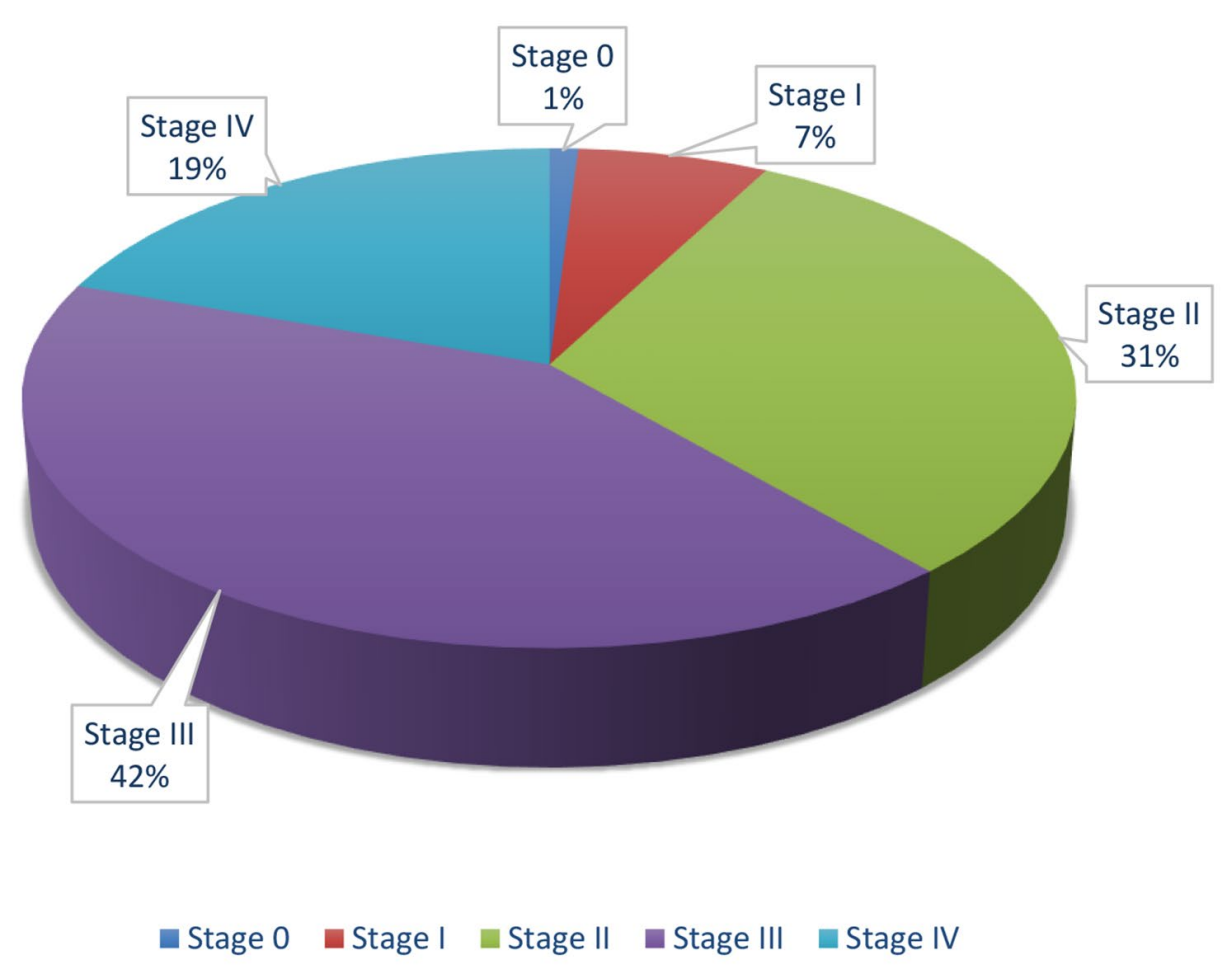
Stages distribution of breast cancer patients in the sample

At the time of diagnosis, only 8% of patients presented with local-stage disease (stages 0 & I), 73% had a regional disease (stages II & III), and 19% had metastatic breast cancer (stage IV) (Fig. 3).

**Fig. 4:**
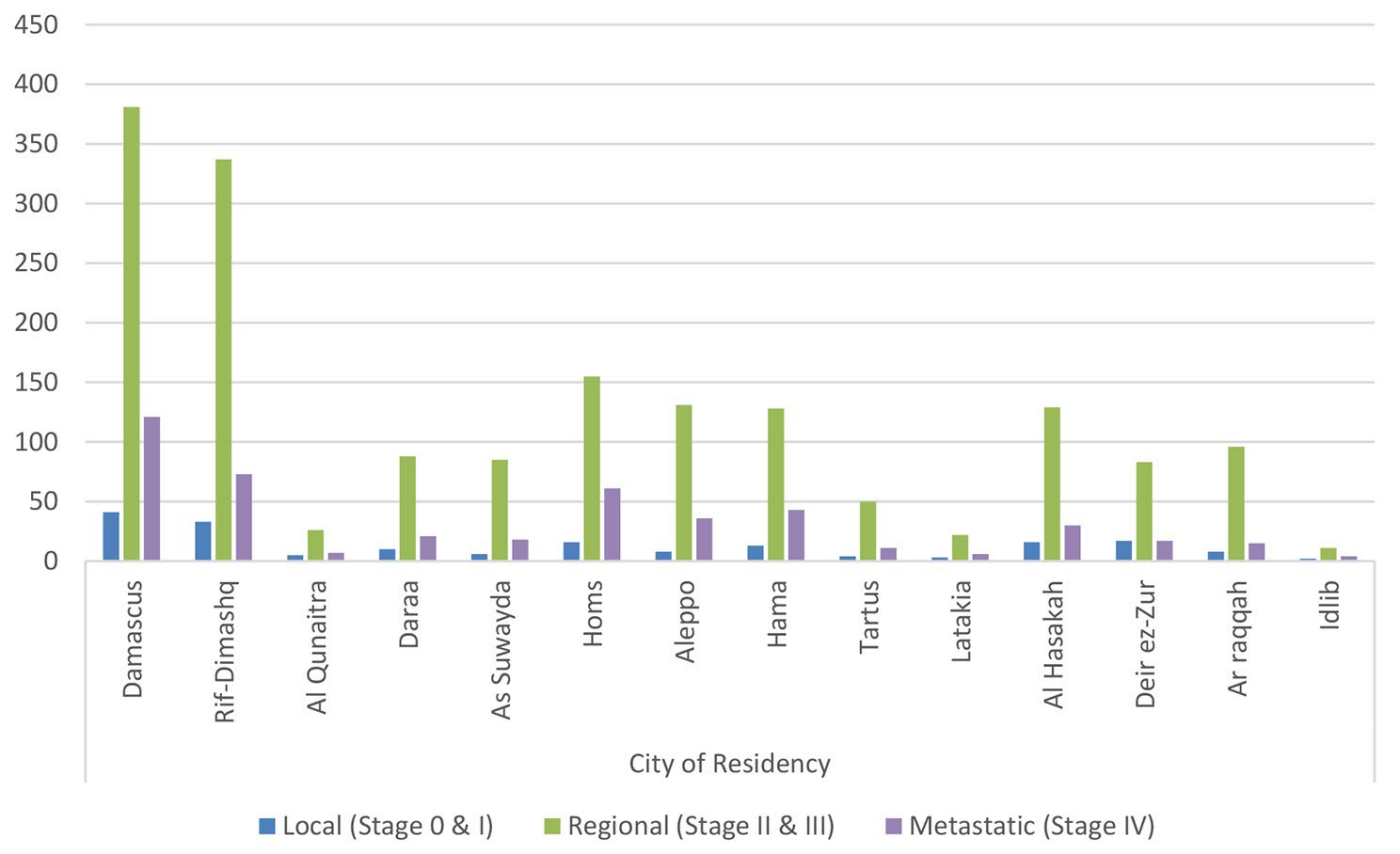
Correlation between patients’ city of residency and disease stage at diagnosis (*P* value = 2.4%)

Correlations between disease stage at diagnosis and other variables are shown in Table 3. The data suggest that breast cancer stage seems to be statistically independent of the patient’s marital status, profession, smoking habits, and the number of children. On the other hand, our findings confirm that stage distribution significantly varies among different age groups and cities of residency (Fig. 4). Increased metastatic disease percentage was found in patients between 30 and 39 years and those between 70 and 79 years, compared with other age groups. Additionally, Homs and Hama had metastatic disease rates of 26% and 23%, respectively, which are significantly higher than the average rate in the population (19%).

**Table 3:** Correlation between disease stage and other variables

	Local* n (*%*)	Disease Stage Regional** n (*%*)	Metastatic*** n (*%*)	*P* value**** (%)
**Age**				**< 0.0001**
20–29	4 *(10)*	24 *(75)*	5 *(15)*	
30–39	17 *(6)*	212 *(72)*	65 *(22)*	
40–49	52 *(8)*	544 *(73)*	144 *(19)*	
50–59	51 *(8)*	539 *(74)*	134 *(18)*	
60–69	30 *(8)*	289 *(72)*	80 *(20)*	
70–79	18 *(13)*	97 *(65)*	32 *(22)*	
80–89	10 *(33)*	17 *(57)*	3 *(10)*	
**Marital Status**				30.1
Married	166 *(7)*	*1558 (73)*	408 *(20)*	
Single	15 *(8)*	*145 (70)*	47 *(22)*	
**Profession**				58.3
Had a Job	39 *(9)*	*313 (74)*	70 *(17)*	
Housewife	143 *(8)*	*1397 (72)*	383 *(20)*	
**Habits**				42.3
Smoker	37 *(8)*	298 *(70)*	96 *(22)*	
Non-smoker	127 *(7)*	1338 *(74)*	340 *(19)*	
**Number of Children**				7.3
0	9 *(4)*	131 *(72)*	42 *(24)*	
1–3	67 *(8)*	601 *(72)*	159 *(20)*	
4–6	59 *(7)*	580 *(73)*	152 *(20)*	
7–9	23 *(9)*	190 *(74)*	43 (1*7)*	
≥ 10	8 *(11)*	56 *(73)*	12 *(16)*	
**Method of Diagnosis**				**0.3**
Surgery	106 *(7)*	1130 *(74)*	279 *(19)*	
Biopsy	73 *(9)*	544 *(71)*	149 *(20)*	

* Stage 0 & I

** Stage II & III

*** Stage IV

**** Chi-square test

**Fig. 5:**
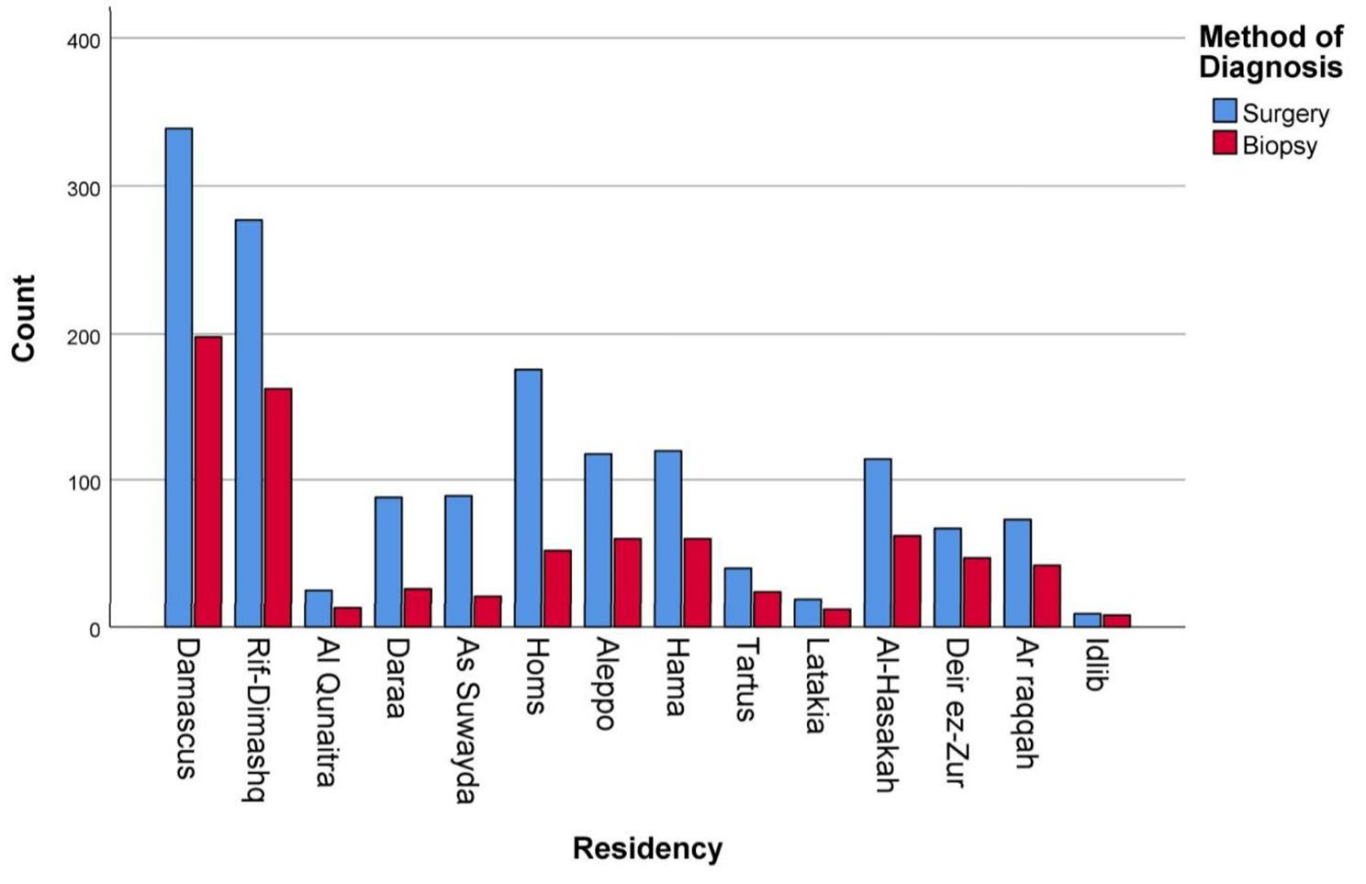
Correlation between patients’ city of residency and disease’s method of diagnosis (*P* value = 0.0002%)

Figure 5 displays how the method of diagnosis distribution differs depending on the patients’ city of residency. Higher surgical biopsies were performed on patients in Homs, Daraa, and As Suwayda. 77%, 77%, and 80% of women, respectively, were diagnosed by surgery in those cities.

## Discussion & conclusion

Breast cancer patients in Syria present with an advanced disease stage compared with other countries. Worldwide, 64% of patients present with a local-stage disease at the time of diagnosis, 27% have a regional disease, and only 6% have metastatic breast cancer at diagnosis [13]. On the other hand, our study revealed that only 8% of Syrian patients presented with local-stage disease (stages 0 & I), 73% had a regional disease at diagnosis (stages II & III), and 19% had metastatic breast cancer (stage IV).

This statistically significant difference results from several causes that could potentially affect the early detection of the disease.

The lack of effective breast cancer screening is the most significant cause of that difference, as less than 5% of the population in our study did screening mammography prior to the breast cancer diagnosis. Factors that might explain limited screening include lack of knowledge and awareness about screening programs, fear of getting diagnosed with cancer, financial restraints, and restricted access to imaging centers for various reasons (long-distance travel might be needed, areas of conflict might prohibit access, and limited numbers of screening centers per capita) [11]. Another factor that plays a role in late detection is the lack of knowledge of the signs and symptoms of breast cancer. Even when the disease manifests clinically, not all patients take the alarming symptoms into consideration [5].

Patients aged between 30 and 39 years are more likely to have aggressive triple-negative tumors compared to older women [14], which results in an advanced disease stage at presentation and explains the high metastatic disease rate in this age group in our study. On the other hand, the increased metastatic disease percentage in patients aged 70–79 years could be explained by the delays in time to diagnosis due to self-neglect and the declining rate of screening among this age group, as there is no sufficient evidence on the benefits and harms of screening in women in their 70s [15–17].

Another worth-mentioning finding is that 64.8% of patients were diagnosed by a surgical procedure (lumpectomy or mastectomy) rather than a biopsy. That could be explained by the lack of knowledge by surgeons and the absence of a multidisciplinary approach to the disease by medical oncology, radiation oncology, and surgical oncology. However, the lack of physicians who can perform imaging-guided biopsies and perhaps the surgeons’ attempt to reduce the financial burden on patients by saving them from doing extra procedures are factors that might explain this malpractice.

Because of the financial restraints and the lack of trained oncologists, there are only two specialized cancer hospitals in Syria. That explains that almost 60% of the study cases came from outside Damascus (the capital of Syria) and its suburbs. In addition, some patients do not trust the local physicians in their villages and tend to go to Damascus for better health care.

Understandably, the Syrian war and its consequences were also major contributing factors that led to the destruction of medical infrastructure in areas of conflict, the fleeing of significant numbers of doctors, and the economic crisis that had a negative impact on the national healthcare budget.

Our retrospective chart review analysis is the first comprehensive review in Syria for female breast cancer patients. It provides epidemiological data and concrete evidence on the disease quality of care based on a large sample obtained from the principal cancer center in Syria (Albairouni University Hospital), which hosts patients from all over the country.

Our findings should be interpreted taking into consideration that most patients treated at Albairouni University Hospital are from low socioeconomic status and that some included cases were diagnosed and treated during the COVID-19 pandemic. Both conditions could affect the higher percentage of advanced-stage disease. In addition, data collection was challenging due to the poor paper documentation of the charts, which can also negatively affect the patients by making their medical information vulnerable to loss. Thus, poor documentation and the high proportion of patients lost to follow-up explain the relatively high number of excluded and missing data.

We recommend simple but effective measures that could help improve patients’ outcomes through early detection and appropriate workup. Decision-makers should work on offering more serious, national campaigns to raise patients’ awareness of the importance of enrolling in screening programs for early detection of the disease. While building other cancer hospitals and imaging centers might be financially challenging, we believe spending the money on early detection and treatment of early-stage breast cancer would be more cost-effective than spending the money on advanced-stage disease. Moreover, healthcare professionals should adopt a multidisciplinary approach to the disease by the different involved specialties to limit the number of unnecessary surgical procedures used to diagnose breast cancer.

In conclusion, our alarming findings and recommendations can serve as the base for future strategies to improve female breast cancer care in Syria.

## Data Availability

All data generated or analyzed during this study are included in this published article.
